# Analysis of Age‐Friendly Service Needs in General Hospital Outpatient Departments Based on the Kano Model: A Cross‐Sectional Study in China

**DOI:** 10.1155/jonm/9802275

**Published:** 2026-11-15

**Authors:** Kangli Wang, Mengjiao Chen, Manjie Yu, Yimiao Xu, Bingjie Fan, Xinlei Chen, Naqin Liu, Yeqin Yang

**Affiliations:** ^1^ School of Nursing Zhejiang Chinese Medical University Hangzhou 310053 Zhejiang China zcmu.edu.cn; ^2^ Sir Run Run Shaw Hospital School of Medicine Zhejiang University Hangzhou 310016 Zhejiang China zju.edu.cn; ^3^ The First Affiliated Hospital of Wenzhou Medical University Wenzhou 325000 Zhejiang China wzhospital.cn

**Keywords:** age-friendly service, general hospital, Kano model, need

## Abstract

**Aim:**

This study aimed to categorise and prioritise the age‐friendly services needs in general hospital outpatient departments.

**Background:**

The traditional outpatient model does not adequately meet the needs of older adults. As global ageing accelerates, advancing age‐friendly outpatient services has grown increasingly urgent.

**Methods:**

We established an item pool for age‐friendly services needs in outpatient departments based on a comprehensive literature review, qualitative interviews and a Delphi expert consultation process. A cross‐sectional survey was conducted between November 2023 and January 2024. Data were collected using a demographic questionnaire and a Kano‐based questionnaire. The attributes and priorities of each need were determined through the satisfaction–importance matrix and the average satisfaction coefficient (ASC).

**Results:**

The item pool comprised 4 dimensions and 43 items related to age‐friendly services. A total of 278 older adults completed the survey. Among the 43 service needs, 18 items were in the improved category, 3 items were in the advantage category, 16 items were in the reserved category and 6 items were in the observation category.

**Conclusions:**

We defined, categorised and prioritised age‐friendly service needs using the Kano model, while identifying differing levels of these needs among older adult outpatients. The satisfaction–importance matrix analysis showed that items under the ‘physical environment’ and ‘information and communication’ were mainly must‐be attributes.

**Implications for Nursing Management:**

The assessment and prioritisation of age‐friendly needs in general hospital outpatient departments enable managers to systematically optimise age‐friendly services, pinpoint key areas for improvement and adopt targeted management strategies to achieve continuous quality improvement of age‐friendly outpatient services.

## 1. Introduction

Demands for geriatric outpatient services are rising steadily [1], yet current systems fail to meet the needs of older adults. According to the World Health Organisation (WHO), the global population aged 60 and above is projected to reach 1.4 billion by 2030. This demographic shift has brought a growing burden of age‐related conditions, characterised by multimorbidity and high chronic disease prevalence [2–4]. Correspondingly, the medical service utilisation rate among older adults is significantly higher than that of the general population [5]. Although Chinese general hospitals have made continuous progress in optimising outpatient services in recent years, they still face numerous problems [6–8], including complex medical processes, insufficient age‐friendly facilities and a distinct ‘digital divide’. These drawbacks greatly impair older adults’ medical experiences, weaken their willingness to seek medical care actively and ultimately undermine their ability to conduct self‐health management [9]. Thus, it is urgently necessary to construct an age‐friendly outpatient environment and optimise service delivery systems for older adults [10].

Age‐friendly practices have become a central global concern. Age‐friendly services refer to approaches designed for the unique needs of older adults, which play a key role in optimising their health and enhancing hospital services [11]. In the United States, reforms have focused on several dimensions, including care processes, organisational support, emotional and behavioural environments, clinical care and research ethics and physical environments [12]. Similarly, Iran has emphasised environmental safety, transportation accessibility and respectful staff‐patient interactions to promote age‐friendly care [13]. In mainland China, a top‐down policy framework has been established and progressively implemented [14]. For example, Beijing launched a 2017 action plan to build age‐friendly hospitals, focusing on key areas: age‐friendly culture, management, service and environment.

Despite the widespread advocacy of age‐friendly policies, a critical gap remains: There is a lack of research exploring age‐friendly outpatient services from the perspective of older adults’ needs. Existing studies have primarily focused on the importance of such services and the challenges encountered during implementation [15, 16]. As needs form the foundation for service system development, only need‐orientated services can be properly targeted and thus effectively enhance older adults’ satisfaction. Prioritising the needs for age‐friendly outpatient services can further identify the most urgently required services, providing clear priority guidance for outpatient managers in resource allocation.

The Kano model, developed by Professor Noriaki Kano of the Tokyo Institute of Technology, is a two‐dimensional modelling tool for classifying and prioritising customer needs [17]. Originally developed for quality management, the Kano model has also been applied to healthcare service research [18–20]. Previous studies have used this model to explore patients’ needs for specific outpatient services, such as medical escort services and diagnostic procedures [21, 22]. The model categorises service needs into five types based on the relationship between service performance and user satisfaction: must‐be attributes (M), attractive attributes (A), indifferent attributes (I), one‐dimensional attributes (O) and reverse attributes (R). In the satisfaction–importance matrix, these needs are further grouped into four areas: Advantage Area I, Improved Area II, Observation Area III and Reserved Area IV. In addition, the average satisfaction coefficient (ASC) is introduced to rank the importance of needs within each quadrant. In this study, the Kano model was used to identify and prioritise older adults’ needs for age‐friendly outpatient services. The findings may help outpatient managers develop targeted improvement strategies by ensuring essential must‐be attributes, improving one‐dimensional attributes and considering attractive attributes, with priority given to needs ranked more highly within each quadrant. These efforts may help outpatient managers better address older adults’ needs for age‐friendly services and enhance the quality of age‐friendly outpatient care.

## 2. Design and Methods

### 2.1. Study Design and Participants

This cross‐sectional study was conducted in the outpatient departments of 13 tertiary general hospitals in Zhejiang, Sichuan and Chongqing, China, from November 2023 to January 2024. Convenience sampling was used to select older adults. The inclusion criteria for participants were as follows: (1) age ≥ 60 years; (2) outpatients or outpatient‐referred patients; (3) can communicate effectively in Mandarin; and (4) have no cognitive impairment. The study was approved by the Institutional Review Board of Wenzhou Medical University, China (no. KY2022‐167). All participants provided written informed consent. They were also informed that they could withdraw from the study at any time.

### 2.2. Sample Size Calculation

Following Kendall’s approach for descriptive studies [23], the sample size was estimated as *N* = *n* × (5–10) × (1 + 10%). Chapman and Callegaro suggested a sample size of at least 200 respondents for Kano model studies [24]. Since there were 43 items in the study, the minimum required sample size was calculated as *N* = (43 × 5) × (1 + 10%) = 237. In the end, this study had 291 participants. ‘*N*’ represents the sample size, and ‘*n’* represents the number of questionnaire items.

### 2.3. Development of an Item Pool for Age‐Friendly Service Needs in General Hospital Outpatient Departments

An item pool for assessing age‐friendly service needs in general hospital outpatient departments was developed through previous literature review, qualitative research and Delphi expert consultation. An initial literature search was conducted across PubMed, Embase, CINAHL, Scopus, Web of Science, The Cochrane Library, China National Knowledge Infrastructure (CNKI), VIP Database, Wanfang Database, Chinese Biomedical Literature Database (CBM) and Taiwan Academic Database using keywords such as ‘age‐friend∗, elder‐friend∗, senior‐friend∗’; ‘hospital, patient, health care, health system’. The literature search focused on age‐friendly service measures, older adults’ needs for age‐friendly services and the standards, frameworks and indicators for age‐friendly hospital development. A total of 34 relevant studies were included to inform the development of a preliminary framework and a pool of candidate items.

Then, in‐depth interviews were conducted with 17 older adults to further explore their needs for age‐friendly outpatient services. Participants were assigned sequential identification codes (N1–N17). Audio recordings were transcribed verbatim within 24 h of each interview and checked by a second researcher with relevant research experience. The transcripts were analysed using conventional content analysis. Meaningful units related to age‐friendly service needs were identified and inductively coded, and similar codes were grouped into categories through team discussions. Data saturation was considered achieved when no substantially new needs emerged. The identified needs were then compared with the literature‐derived item pool. Newly identified needs were developed into candidate items, while overlapping items were merged or refined. The resulting items were incorporated into the preliminary item pool for Delphi expert consultation.

Subsequently, a two‐round Delphi consultation was conducted with 20 experts in geriatric nursing. All experts had more than 10 years of professional experience, a bachelor’s degree or higher and a senior professional title. Before the consultation, the consensus and item‐retention criteria were prespecified. A CV ≤ 0.25 was considered acceptable, and Kendall’s W was used to assess overall agreement among experts, with statistical significance tested using the chi‐square test. For item retention, the cutoff values for Mj and Kj were calculated as their respective means minus standard deviations, whereas the cutoff value for CV was calculated as its mean plus standard deviation. Items were retained when Kj and Mj exceeded their respective cutoff values and CV was below its cutoff value. Items meeting none of these criteria were deleted. For items meeting only one or two criteria, the research team made the final decision based on their importance, feasibility and expert comments.

The expert activity coefficients were 80% and 95% in the two rounds, and the authority coefficients were 0.888 and 0.887, respectively. The indicators generally received high importance scores and full‐score rates, with relatively low SDs and CVs. Kendall’s W increased from 0.200 to 0.250 (*p* < 0.001). Following the second round and subsequent discussion within the research team, the consultation was concluded. The final item pool comprised 43 items across four dimensions (Table 1).

**TABLE 1:** Item pool for age‐friendly service needs in the general hospital outpatient department.

Dimensions	Items	Items number
Physical environment	Having public transportation near the hospital	A1
	Providing shuttle bus service	A2
	Having temporary pick‐up/drop‐off zones	A3
	Providing ample parking within the hospital	A4
	Having a comfortable overall environment	A5
	Preventing ground slips	A6
	Centralising diagnostic and treatment areas	A7
	Providing walking aids	A8
	Facilitating easily identifiable signage	A9
	Having sufficient seating in rest areas	A10
	Providing barrier‐free access	A11
	Providing barrier‐free restrooms	A12

Information and communication	Maintaining amiable communication attitude	B1
	Ensuring comprehensible staff communication	B2
	Listening to patient concerns and needs actively	B3
	Disclosing disease‐related information proactively	B4
	Encouraging full‐process patient engagement	B5
	Having an easy‐to‐understand medical treatment guide	B6
	Promoting accessible medical services	B7
	Delivering disease care updates digitally	B8
	Delivering geriatric‐focused health education digitally	B9
	Ensuring consistent diagnostic information	B10
	Protecting personal privacy	B11

Medical nursing professional services	Operating nighttime outpatient services	C1
	Providing geriatric joint outpatient services	C2
	Delivering specialised geriatric outpatient services	C3
	Providing geriatric comprehensive assessment services	C4
	Offering pharmaceutical counselling services	C5
	Delivering nutritional counselling services	C6
	Providing geriatric rehabilitation services	C7
	Providing palliative care services	C8
	Providing continuous nursing care	C9

Medical process services	Guiding medical services	D1
	Providing volunteer services	D2
	Accompanying throughout treatment	D3
	Assisting in using the self‐service machine	D4
	Providing staffed one‐stop service counters	D5
	Prioritising geriatric care coordination	D6
	Enabling online registration	D7
	Facilitating proxy registration for older adults	D8
	Ensuring punctuality of outpatient services	D9
	Offering luggage storage	D10
	Providing mailing services	D11

### 2.4. The Kano Model

#### 2.4.1. Demand Attributes

According to the Kano model, the positive and negative responses to each questionnaire item can be employed to classify its attribute type. This study adopted the maximum frequency method to categorise all service needs (see Table 2).

**TABLE 2:** Classification of demand attributes in the Kano model.

**Negative question**						
**Positive question**	**Items**	**Likes**	**Should be**	**Does not matter**	**Can be accepted**	**Dislikes**

	Likes	Q	A	A	A	O
	Should be	R	I	I	I	M
	Does not matter	R	I	I	I	M
	Can be accepted	R	I	I	I	M
	Dislikes	R	R	R	R	Q

*Note:* M: must‐be attribute; O: one‐dimensional attribute; A: attractive attribute; I: indifferent attribute; R: reverse attribute.

#### 2.4.2. The Better–Worse Coefficient and Satisfaction–Importance Matrix

The Kano model introduces the concepts of satisfaction index (SI) and dissatisfaction index (DI) to evaluate the relative importance of different demands. The SI is calculated as SI = (A + O)/(A + O + M + I), and the DI as DI = (−1) × (M + O)/(A + O + M + I). Both indices range in absolute value from 0 to 1, with values closer to 1 indicating a greater impact on satisfaction or importance. A satisfaction–importance matrix was constructed, using |DI| on the horizontal axis and SI on the vertical axis, with the origin (O) defined by the mean values (mean |DI|, mean SI).

#### 2.4.3. ASC Value

The ASC serves as a more intuitive substitute for the better–worse coefficient in reflecting the importance of a requirement and is calculated as ASC = (SI + |DI|)/2. The priority ranking among requirement attributes follows the order M > O > A > I > R, and the priority ranking in the same quadrant is based on the ASC index.

### 2.5. Measures

#### 2.5.1. Demographic Information Questionnaire

Informed by the literature review, we designed a questionnaire to collect the participants’ demographic information that included sex, age, place of residence, education level, marital status, monthly personal income and number of chronic diseases.

#### 2.5.2. Age‐Friendly Service Needs

The scale comprised 43 items across 4 dimensions: physical environment, information and communication, medical nursing professional services and medical process services. Each item was designed using the Kano model’s bidirectional questioning approach, featuring paired positive and negative statements. The response options were classified into five categories: like, should be, does not matter, can be accepted and dislikes. Before the formal survey, a pilot study was carried out with 50 older adults at general hospitals. The scale has good reliability and validity: a content validity index (S‐CVI) of 0.86, an item‐level content validity index (I‐CVI) ranging from 0.833 to 1.00, and Cronbach’s *α* values of 0.893 for the positive questionnaire and 0.913 for the negative questionnaire.

### 2.6. Data Collection

After providing informed consent, participants completed the questionnaires. Research team members explained the purpose and completion procedure to the participants. Following collection, each questionnaire was reviewed for authenticity and completeness. Those with clearly unreasonable responses, including all answers being identical or illogical responses, were excluded.

### 2.7. Statistical Analysis

Statistical analyses were performed using SPSS 25.0. Descriptive statistics summarised the baseline characteristics, with categorical data presented as frequencies and percentages. Following Kano methodology, service demand attributes were classified accordingly. The SI, DI and ASC were calculated for each service item, and a satisfaction–importance matrix was constructed. Service priorities were determined by integrating ASC values. Bootstrap resampling (5000 iterations) was used to estimate the 95% confidence intervals (CIs) for the SI, |DI| and ASC values.

## 3. Results

### 3.1. Demographic Information

A total of 291 older adults participated in the study. However, 13 invalid questionnaires (4.5%) were excluded, representing an effective response rate of 95.5%. The mean age of the participants was 68.58 ± 6.03 years, with the majority (60.4%) aged 60–69 years. Of the participants, 170 (61.2%) were male and 108 (38.8%) were female; 145 (52.2%) lived in rural areas, while 133 (47.8%) lived in urban areas. Additional demographic characteristics are presented in Table 3.

**TABLE 3:** General information of participants (*n* = 278).

Variables		*n*	%
Sex	Male	170	61.2
	Female	108	38.8

Age (years)	60–69	168	60.4
	70–79	94	33.8
	≥ 80	16	5.8

Place of residence	Urban	133	47.8
	Rural	145	52.2

Education level	Primary school and below	133	47.8
	Secondary school	119	42.8
	Junior college and above	26	9.4

Marital status	Married	268	96.4
	Widowed	10	3.6

Number of children	0	1	0.4
	1	70	25.2
	2	101	36.3
	≥ 3	106	38.1

Cross‐regional treatment	Yes	43	15.5
	No	235	84.5

Monthly personal income (RMB)	≤ 2000	94	33.8
	2001–4000	67	23.7
	4001–6000	46	16.5
	≥ 6000	71	25.5

Number of chronic diseases	0	22	7.9
	1	88	31.7
	2	84	30.2
	≥ 3	84	30.2

Ways to enter the outpatient department	Walking	251	90.3
	Crutches/wheelchair	27	9.7

Reasons for choosing the hospital	Advanced medical technology	164	59.0
	Conveniently located	45	16.2
	Quality service environment	10	3.6
	Personal recommendation	48	17.3
	Other	11	4.0

Family‐accompanied	Yes	221	79.5
	No	57	20.5

Transportation modes	Driven by family member	147	52.9
	Public transit	45	16.2
	Hailing a taxi	38	13.7
	Self‐driving	37	13.3
	Walking	11	4.0

Registering independently	Yes	90	32.4
	No	188	67.7

Operating self‐service machines	Yes	37	13.3
	No	241	86.7

### 3.2. Attributes and Better–Worse Index of Age‐Friendly Service Needs in Outpatient

Among the 43 age‐friendly service needs assessed in general hospital outpatient departments, 8 items (18.6%) were identified as must‐be attributes (M), 32 items (74.4%) as attractive attributes (A) and 3 items (7.0%) as indifferent attributes (I). No one‐dimensional attributes (O) were identified. The SI values ranged from 0.396 to 0.741, while the |DI| values ranged from 0.097 to 0.615. ‘Providing geriatric joint outpatient services’ (C2) demonstrated the highest SI value (0.741), whereas ‘Maintaining amiable communication attitude’ (B1) showed the highest |DI| value (0.615) (see Table 4).

**TABLE 4:** Needs attribute classification and ASC ranking for each service.

Quadrant/attribute	Items number	Frequency					Kano attributes	SI	|DI|	ASC	ASC order
		A	O	M	I	R					
Quadrant I/one‐dimensional attribute	A7	143	56	29	50	0	A	0.716	0.306	0.511	1
	D2	141	24	68	45	0	A	0.594	0.331	0.462	2
	A4	107	51	39	81	0	A	0.568	0.324	0.446	3

Quadrant II/attractive attribute	C2	173	33	24	48	0	A	0.741	0.205	0.473	1
	D5	174	29	28	47	0	A	0.730	0.205	0.468	2
	D9	145	28	54	51	0	A	0.622	0.295	0.459	3
	D6	144	29	38	67	0	A	0.622	0.241	0.432	4
	B7	139	32	34	73	0	A	0.615	0.237	0.426	5
	D4	136	24	50	68	0	A	0.576	0.266	0.421	6
	A10	124	36	37	81	0	A	0.576	0.263	0.419	7
	C1	159	24	25	70	0	A	0.658	0.176	0.417	8
	C4	164	23	20	71	0	A	0.673	0.155	0.414	9
	C3	174	20	14	70	0	A	0.698	0.122	0.410	10
	C9	170	16	17	75	0	A	0.669	0.119	0.394	11
	A2	171	16	10	81	0	A	0.673	0.094	0.383	12
	C6	153	21	16	88	0	A	0.626	0.133	0.379	13
	C7	152	22	15	89	0	A	0.626	0.133	0.379	13
	D11	164	19	7	88	0	A	0.658	0.094	0.376	15
	D3	150	23	6	99	0	A	0.622	0.104	0.363	16
	B9	143	16	19	100	0	A	0.572	0.126	0.349	17
	C8	144	17	16	101	0	A	0.579	0.119	0.349	17

Quadrant III/indifferent attribute	B5	123	25	56	74	0	A	0.532	0.291	0.412	1
	B6	117	30	47	84	0	A	0.529	0.277	0.403	2
	C5	119	26	46	87	0	A	0.522	0.259	0.390	3
	B8	120	20	48	90	0	A	0.504	0.245	0.374	4
	A1	110	29	33	106	0	A	0.500	0.223	0.362	5
	D10	105	14	13	146	0	I	0.428	0.097	0.263	6

Quadrant IV/must‐be attribute	B1	67	59	112	40	0	M	0.453	0.615	0.534	1
	D7	86	43	109	40	0	M	0.464	0.547	0.505	2
	B3	62	49	121	46	0	M	0.399	0.612	0.505	3
	A6	84	45	103	46	0	M	0.464	0.532	0.498	4
	D8	91	46	93	48	0	M	0.493	0.500	0.496	5
	B11	63	48	115	52	0	M	0.399	0.586	0.493	6
	B2	104	44	78	52	0	A	0.532	0.439	0.486	7
	B10	104	44	78	52	0	M	0.439	0.522	0.480	8
	A9	91	43	89	55	0	A	0.482	0.475	0.478	9
	A5	99	46	67	66	0	A	0.522	0.406	0.464	10
	B4	89	33	101	55	0	M	0.439	0.482	0.460	11
	D1	112	27	81	58	0	A	0.500	0.388	0.444	12
	A3	111	40	49	78	0	A	0.543	0.320	0.432	13
	A12	89	32	75	82	0	A	0.435	0.385	0.410	14
	A11	79	31	83	85	0	I	0.396	0.410	0.403	15
	A8	75	40	62	101	0	I	0.414	0.367	0.390	16

### 3.3. Satisfaction–Importance Matrix and ASC of Age‐Friendly Service Needs

In the overall sample, the 43 service attributes were distributed across the four quadrants of the satisfaction–importance matrix. Three items (7.0%) were classified into Quadrant I, with A7 having the highest ASC. Eighteen items (41.9%) were classified into Quadrant II, with C2 having the highest ASC. Six items (14.0%) were classified into Quadrant III, with B5 having the highest ASC. The remaining 16 items (37.2%) were classified into Quadrant IV, with B1 having the highest ASC (Figure 1 and Table 5). The bootstrap‐estimated 95% CIs for the SI, |DI| and ASC values are presented in the Supporting Information (available (here)).

**FIGURE 1:**
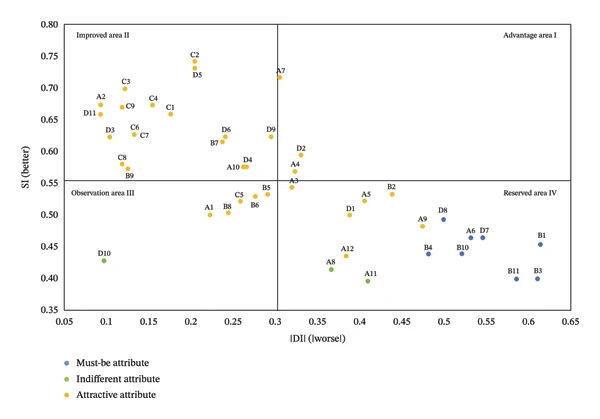
Satisfaction–importance matrix for each service.

**TABLE 5:** Comparison of needs attribute results.

Demand attributes	Traditional Kano classification results	Satisfaction–importance matrix classification results
Must‐be attribute	A6	A3 A5 A6 A8 A9 A11 A12
	B1 B3 B4 B10 B11	B1 B2 B3 B4 B10 B11
	D7 D8	D1 D7 D8

One‐dimensional attribute	/	A4 A7
		D2

Attractive attribute	A1 A2 A3 A4 A5 A7 A9 A10 A12	A2 A10
	B2 B5 B6 B7 B8 B9	B7 B9
	C1 C2 C3 C4 C5 C6 C7 C8 C9	C1 C2 C3 C4 C6 C7 C8 C9
	D1 D2 D3 D4 D5 D6 D9 D11	D3 D4 D5 D6 D9 D11

Indifferent attribute		A1
	A8 A11	B5 B6 B8
	D10	C5
		D10

## 4. Discussion

This study employed the Kano model and the satisfaction–importance matrix to categorise age‐friendly outpatient service needs and further introduced the ASC value to prioritise needs within each quadrant. The results indicated that these needs were primarily clustered into must‐be, attractive and indifferent attributes, with no one‐dimensional attributes identified. Consistent with previous studies [18, 21, 25], must‐be and attractive attributes are generally regarded as the core components of medical service demands, whereas the absence of one‐dimensional attributes appears relatively unusual. A plausible explanation is that current age‐friendly outpatient services remain underdeveloped, and older adults have limited awareness and understanding of such services. Furthermore, while most previous Kano model‐based studies have focused on inpatient or community settings, this study explored age‐friendly services in the outpatient setting and supporting the Kano model with the ASC value. These findings not only expand the application of the Kano model in the health domain but also provide targeted evidence for optimising age‐friendly outpatient services.

In this study, all 8 must‐be attribute items and 8 items from other categories were located in the fourth quadrant. The fourth quadrant typically represents reserved attributes, corresponding to basic service needs. Their absence leads to strong dissatisfaction, whereas their presence only satisfies basic expectations. In the physical environment dimension, safety and accessibility measures including nonslip floors (A6), barrier‐free access (A11) and 5 other services are fundamental prerequisites for accessing healthcare. Age‐related physiological decline increases older adults’ vulnerability, making it more difficult to meet fundamental physiological and safety needs [26, 27]. An age‐friendly physical environment supports the satisfaction of these needs and is thus fundamental to geriatric care. Predebon and colleagues reported that nearly one‐third of older adults had mobility impairments, and inadequate age‐friendly facilities constitute a major barrier to healthcare access [28, 29]. In the information and communication dimension, maintaining amiable communication attitude (B1), disclosing disease‐related information proactively (B4), and 4 other services are core components of effective doctor–patient interaction. Due to heightened psychological sensitivity, older adults value both subtle emotional expressions and reliable health information access [30, 31]. Previous studies have shown that effective health communication and improved understanding of health management greatly enhance positive healthcare experiences [32]. In the medical process service dimension, online registration (D7), proxy registration (D8) and medical guidance services (D1) are key measures to address older adults’ difficulty accessing medical care. Registration is the first step in seeking care and is crucial to optimising older adults’ medical experience. However, the digital transformation of healthcare services presents significant challenges for older adults in adapting [33, 34]. Only 50 participants (18.0%) in our study could independently complete online registration. In conclusion, healthcare administrators should prioritise must‐be attributes, focusing on enhancing information and communication, optimising physical environment and streamlining medical process services. This integrated strategy will help construct a more comprehensive and effective age‐friendly service system.

In this study, no service items were categorised as one‐dimensional attributes, but three items—providing ample parking within the hospital (A4), centralising diagnostic and treatment areas (A7) and providing volunteer services (D2)—fell into the first quadrant. Items in this quadrant exert significant influence on both importance and satisfaction, making them key priorities for optimising age‐friendly services. During the survey, a notably high tolerance was observed among older adults, often choosing responses like ‘Does not matter’ or ‘Can be accepted’ when services were unavailable. This response pattern may be related to the traditional Confucian emphasis on moderation among the Chinese elderly population, which might suppress the explicit expression of their healthcare needs [35, 36]. In addition, this discrepancy may stem from the traditional Kano model’s methodological limitations, which categorises items based on the highest response frequency and fails to fully capture the psychosocial and cultural factors [37]. Although older adults showed high adaptability, this does not mean that their needs were fully met. Therefore, we recommend integrating the traditional Kano model with a satisfaction–importance matrix to establish a multidimensional framework for age‐friendly care [38]. Healthcare administrators should proactively identify and address unexpressed service expectations that could significantly improve older adults’ healthcare experiences.

In this study, 32 service needs were categorised as attractive attributes, of which 56.25% (18/32) fell into the second quadrant. Meeting these service needs can significantly enhance older adults’ satisfaction. Medical nursing professional services, including geriatric joint outpatient services (C2), specialised geriatric care services (C3) and 6 other services fell into the second quadrant. Older adults tend to have complex and diverse health needs and rely heavily on specialised geriatric care to improve health outcomes. A cross‐sectional study reported a 73.2% comorbidity rate among this population, highlighting the need to refine these specialised services [39]. Targeted geriatric services help address older adults’ unique health challenges, while some services encounter practical obstacles such as insufficiently prominent speciality features, inadequate interprofessional collaboration and excessive resource allocation demands [40, 41]. The study also found that convenient medical process services and supportive physical environment services such as punctuality of outpatient services (D9) and shuttle bus services (A2) were in the second quadrant, reflecting older adults’ unmet demands for streamlined and humanised care. Consistent with previous research, registration and wait time are critical factors in older adults’ overall healthcare experience, with prolonged waits diminishing patients’ willingness to revisit [42, 43]. Similarly, shuttle bus services and sufficient seating accommodate patients’ limited mobility and endurance, fostering a sense of being cared for and valued [44, 45]. Healthcare administrators can use these findings to enhance service quality, for example, expanding access to specialised geriatric services, refining streamlined medical processes and upgrading supportive physical environment.

In this study, all 3 indifferent attribute items and 3 items from other attribute categories fell into the third quadrant, including having public transportation near the hospital (A1) and 5 other services. These items exerted limited influence on both patient satisfaction and dissatisfaction. This finding may reflect the tendency to prioritise core clinical services and service efficiency over such supportive services [46, 47]. Furthermore, outpatient settings have distinct characteristics: Patients typically experience short and progressively reduced stay durations, which may reduce the perceived value of supporting services [48]. Consequently, medical institutions need not devote substantial resources to these indifferent attribute services. Instead, they should allocate resources to services that significantly impact older adults’ satisfaction, thereby optimising resource utilisation, reducing unnecessary costs and sustaining high service quality.

The differences between the Kano model and the satisfaction–importance matrix primarily stem from the following logical perspectives. The Kano model focuses on the universal classification of user needs, offering general applicability. In contrast, the satisfaction–importance matrix analysis is entirely grounded in our selected sample of older adults. It can intuitively demonstrate the relative importance and satisfaction levels of different needs within this population, thereby effectively pinpointing important yet unmet needs. Therefore, outpatient managers should exercise caution when applying these matrix analysis results. When addressing specific populations in the future, such as rural older adults or highly educated older adults, it will be necessary to reconduct the satisfaction–importance matrix analysis for these groups.

Through ASC‐based ranking analysis, this study found significant priority disparities for age‐friendly service demands in general hospital outpatient departments. Among the must‐be attributes, ‘Maintaining amiable communication attitude’ (B1, ASC = 0.534) ranked highest, highlighting the key role of humanistic care in healthcare services. We recommend systematically integrating patient‐centred communication skills into healthcare providers’ core training, with a focus on building their capacity to express respect and understanding through empathetic language and actions [47, 49]. Among indifferent attributes, ‘Luggage storage services’ (D10, ASC = 0.263) had the lowest improvement value. This likely is related to the typically short outpatient visits, high patient turnover and the large share of local residents in the patient cohort, which reduces reliance on such services [50]. It should be noted that demand attributes in the Kano model are inherently dynamic. As medical services improve, current indifferent attributes may shift to must‐be or attractive attributes over time [37]. Therefore, while focusing on current priorities, administrators should take a forward‐looking perspective to anticipate evolving expectations, ensuring the ongoing relevance and effectiveness of age‐friendly service systems.

## 5. Limitations

This study has several limitations. First, convenience sampling resulted in a relatively younger sample, and the sample sizes varied substantially across the 13 participating hospitals. These factors may have limited the comparability of findings across sites and the generalisability of the results. Second, the quantitative design may not have fully captured the multidimensional needs of older adults for age‐friendly services. Future research should adopt mixed‐methods designs, include participants from multiple regions and recruit more balanced samples across sites and age groups to gain a more comprehensive understanding of these needs.

## 6. Conclusions

This study developed an item pool of 43 age‐friendly outpatient service items across 4 dimensions and applied the Kano model combined with the ASC to classify and prioritise these needs. The satisfaction–importance matrix analysis revealed that items in the dimensions of ‘physical environment’ and ‘information and communication’ were primarily must‐be attributes, whereas those in ‘medical nursing professional services’ and ‘medical process services’ were mainly attractive attributes. These findings highlight that healthcare administrators should dynamically assess service needs by taking into account the cultural backgrounds and individual differences of older adults, thereby providing a basis for optimising age‐friendly outpatient services.

## 7. Implications for Nursing Management

The findings of this study can provide clear guidance for outpatient managers in identifying and prioritising age‐friendly service needs. Under resource constraints, managers should prioritise must‐be attributes, monitor evolving service needs for emerging one‐dimensional attributes and optimise attractive attributes to enhance older adults’ service experience. Notably, the results of the satisfaction–importance matrix reflect the needs and priorities of the specific study population and should therefore be generalised to other populations or settings with caution. Future studies could assign weights to each service items and develop a standardised evaluation system to support the quantitative assessment and continuous improvement of age‐friendly outpatient services.

## Author Contributions

Kangli Wang: writing–original draft preparation and methodology. Mengjiao Chen: writing–original draft preparation and investigation. Manjie Yu: methodology, data curation, and formal analysis. Yimiao Xu: methodology and data curation. Bingjie Fan: investigation and data curation. Xinlei Chen: methodology and data curation. Naqin Liu: methodology and data curation. Yeqin Yang: writing–reviewing and editing, resources, methodology, supervision, and conceptualization.

## Funding

This work was supported by the Humanities Nursing Research Project of the Humanities Nursing Professional Committee, China Life Care Association (grant no. RW2024YB04).

## Conflicts of Interest

The authors declare no conflicts of interest.

## Supporting Information

Additional supporting information can be found online in the Supporting Information section.

## Supporting information


**Supporting Information 1** Table S1: Summary of literature review extracting needs of age‐friendly services.

**Supporting Information 2** Table S2: Themes and subthemes identified from interviews.

**Supporting Information 3** Table S3: Characteristics of the participating hospitals and recruitment of participants.

**Supporting Information 4** Table S4: Bootstrap 95% confidence intervals of SI, |DI| and ASC for each Kano item.

## Data Availability

The data that support the findings of this study are available from the corresponding author upon reasonable request.
