# Supplementary material for: Analysis of Age‐Friendly Service Needs in General Hospital Outpatient Departments Based on the Kano Model: A Cross‐Sectional Study in China
Source: J Nurs Manag. 2026 Nov 15;2026:9802275. doi: 10.1155/jonm/9802275 (PMC13770278; doi:10.1155/jonm/9802275)
Supplement: Supplementary file 1 [file JONM-2026-9802275-s001.docx]

**TABLE S1 Summary of Literature Review Extracting Needs of Age-Friendly Services**

| **Dimensions** | **Items** | **Items number** |
| --- | --- | --- |
| Physical environment | Providing convenient public transport access to the hospital | Ⅲ |
|  | Providing shuttle bus service | Ⅲ |
|  | Having temporary pick-up/drop-off zones | Ⅰ, Ⅲ |
|  | Providing a dedicated entrance for older adults | Ⅰ |
|  | Having a comfortable overall environment | ⅠⅡ, Ⅲ |
|  | Centralising diagnostic and treatment areas | Ⅰ, Ⅱ, Ⅲ |
|  | Providing barrier-free access | Ⅰ, Ⅲ |
|  | Providing walking aids | Ⅰ, Ⅱ Ⅲ |
|  | Providing assistive devices for older adults | Ⅰ, Ⅲ |
|  | Facilitating easily identifiable signage | Ⅰ, Ⅱ, Ⅲ |
|  | Having sufficient seating in rest areas | Ⅰ, Ⅲ |
|  | Providing barrier-free restrooms | Ⅰ, Ⅲ |
|  | Facilitating convenient dining | Ⅱ |
| Information and communication | Maintaining amiable communication attitude | Ⅰ, Ⅱ, Ⅲ |
|  | Ensuring comprehensible staff communication | Ⅲ |
|  | Listening to patient concerns and needs actively | Ⅲ |
|  | Disclosing disease-related information proactively | Ⅰ, Ⅱ, Ⅲ |
|  | Encouraging full-process patient engagement | Ⅰ, Ⅲ |
|  | Explaining test precautions in detail | Ⅲ |
|  | Protecting personal privacy | Ⅱ |
|  | Posting geriatric health education in waiting areas | Ⅰ, Ⅲ |
|  | Having an easy-to-understand medical treatment guide | Ⅲ |
|  | Promoting accessible medical services | Ⅰ, Ⅲ |
|  | Wearing staff identification badges | Ⅲ |
| Medical nursing professional services | Delivering specialised geriatric outpatient services | Ⅰ, Ⅱ, Ⅲ |
|  | Providing geriatric comprehensive assessment services | Ⅰ, Ⅲ |
|  | Offering pharmaceutical counselling services | Ⅰ, Ⅲ |
|  | Delivering nutritional counselling services | Ⅲ |
|  | Providing geriatric rehabilitation services | Ⅰ, Ⅲ |
|  | Providing palliative care services | Ⅰ, Ⅲ |
|  | Providing continuous nursing care | Ⅰ, Ⅲ |
| Medical technical and auxiliary services | Guiding medical services | Ⅰ, Ⅲ |
|  | Providing volunteer services | Ⅰ, Ⅲ |
|  | Assisting in using the self-service machine | Ⅰ, Ⅱ, Ⅲ |
|  | Providing a platform for older volunteer participation | Ⅰ, Ⅲ |
|  | Providing staffed one-stop service counters | Ⅰ |
|  | Providing dedicated service windows for older adults | Ⅰ, Ⅲ |
|  | Prioritising geriatric care coordination | Ⅰ, Ⅱ, Ⅲ |
|  | Facilitating outpatient registration | Ⅰ, Ⅱ |
|  | Enabling online registration | Ⅰ, Ⅲ |
|  | Facilitating proxy registration for older adults | Ⅰ, Ⅲ |
|  | Facilitating outpatient examinations | Ⅰ |
|  | Facilitating outpatient test report retrieval | Ⅰ |
|  | Facilitating outpatient payment | Ⅲ |
|  | Facilitating outpatient medication pick-up | Ⅰ |
|  | Rationalising medical service fees | Ⅱ, Ⅲ |
|  | Rationalising queue times at service counters | Ⅱ |
|  | Ensuring punctuality of outpatient services | Ⅲ |
|  | Providing luggage storage and mailing services | Ⅰ, Ⅲ |
| **Note: Ⅰ, implementation of age-friendly service measures in general hospital outpatient departments; Ⅱ, older adults' needs for age-friendly outpatient services; Ⅲ, standards, frameworks, and indicators for age-friendly hospital construction.** | | |
