# Supplementary material for: Analysis of Age‐Friendly Service Needs in General Hospital Outpatient Departments Based on the Kano Model: A Cross‐Sectional Study in China
Source: J Nurs Manag. 2026 Nov 15;2026:9802275. doi: 10.1155/jonm/9802275 (PMC13770278; doi:10.1155/jonm/9802275)
Supplement: Supplementary file 2 [file JONM-2026-9802275-s002.docx]

**TABLE S2 Themes and subthemes identified from interviews**

| **Themes** | **Categories** |
| --- | --- |
| Physical environment | Providing ample parking within the hospital |
|  | Having sufficient seating in rest areas |
|  | Centralising diagnostic and treatment areas |
|  | Facilitating easily identifiable signage |
|  | Providing barrier-free restrooms |
| Medical process services | Facilitating convenient registration |
|  | Guiding medical services |
|  | Optimising patient consultation procedures |
|  | Reducing outpatient waiting time |
|  | Providing care during medical visits |
|  | Delivering specialised geriatric outpatient services |
|  | Operating nighttime outpatient services |
| Information and communication | Maintaining amiable communication attitude |
|  | Listening to patient concerns and needs actively |
|  | Disclosing disease-related information proactively |
|  | Ensuring communication effectiveness |
|  | Ensuring consistent diagnostic information |
| Social and support services | Providing volunteer services |
|  | Accompanying throughout treatment |
