# Supplementary material for: Analysis of Age‐Friendly Service Needs in General Hospital Outpatient Departments Based on the Kano Model: A Cross‐Sectional Study in China
Source: J Nurs Manag. 2026 Nov 15;2026:9802275. doi: 10.1155/jonm/9802275 (PMC13770278; doi:10.1155/jonm/9802275)
Supplement: Supplementary file 3 [file JONM-2026-9802275-s003.docx]

**TABLE S3 Characteristics of the participating hospitals and recruitment of participants**

| **Hospital** | **Province** | **Major characteristics** | **Valid sample, n** |
| --- | --- | --- | --- |
| Hospital 1 | Wenzhou, Zhejiang | Tertiary general hospital | 228 |
| Hospital 2 | Jiaxing, Zhejiang | Tertiary general hospital | 14 |
| Hospital 3 | Ningbo, Zhejiang | Tertiary general hospital | 9 |
| Hospital 4 | Taizhou, Zhejiang | Tertiary general hospital | 7 |
| Hospital 5 | Hangzhou, Zhejiang | Tertiary general hospital | 5 |
| Hospital 6 | Hangzhou, Zhejiang | Tertiary general hospital | 5 |
| Hospital 7 | Wenzhou, Zhejiang | Tertiary general hospital | 3 |
| Hospital 8 | Chengdu, Sichuan | Tertiary general hospital | 2 |
| Hospital 9 | Nibo, Zhejiang | Tertiary general hospital | 1 |
| Hospital 10 | Wenzhou, Zhejiang | Tertiary general hospital | 1 |
| Hospital 11 | Shaoxing, Zhejiang | Tertiary general hospital | 1 |
| Hospital 12 | Wenzhou, Zhejiang | Tertiary general hospital | 1 |
| Hospital 13 | Chongqing | Tertiary general hospital | 1 |
