# Supplementary material for: Analysis of Age‐Friendly Service Needs in General Hospital Outpatient Departments Based on the Kano Model: A Cross‐Sectional Study in China
Source: J Nurs Manag. 2026 Nov 15;2026:9802275. doi: 10.1155/jonm/9802275 (PMC13770278; doi:10.1155/jonm/9802275)
Supplement: Supplementary file 4 [file JONM-2026-9802275-s004.docx]

**TABLE S4 Bootstrap 95% confidence intervals of SI, |DI|, and ASC for each Kano item.**

| **Items** | **SI** | **Bootstrap 95% Cl** | **\|DI\|** | **Bootstrap 95% Cl** | **ASC** | **Bootstrap 95% Cl** |
| --- | --- | --- | --- | --- | --- | --- |
| A1 | 0.500 | [0.442-0.558] | 0.223 | [0.176-0.273] | 0.362 | [0.324-0.399] |
| A2 | 0.673 | [0.619-0.727] | 0.094 | [0.061-0.130] | 0.383 | [0.351-0.416] |
| A3 | 0.543 | [0.486-0.601] | 0.320 | [0.266-0.374] | 0.432 | [0.394-0.469] |
| A4 | 0.568 | [0.511-0.629] | 0.324 | [0.270-0.381] | 0.446 | [0.407-0.486] |
| A5 | 0.522 | [0.464-0.580] | 0.406 | [0.349-0.464] | 0.464 | [0.426-0.502] |
| A6 | 0.464 | [0.407-0.522] | 0.532 | [0.475-0.590] | 0.498 | [0.434-0.531] |
| A7 | 0.716 | [0.662-0.770] | 0.306 | [0.252-0.360] | 0.511 | [0.475-0.549] |
| A8 | 0.414 | [0.356-0.475] | 0.367 | [0.309-0.425] | 0.390 | [0.351-0.430] |
| A9 | 0.482 | [0.425-0.543] | 0.475 | [0.417-0.532] | 0.478 | [0.442-0.514] |
| A10 | 0.576 | [0.518-0.633] | 0.263 | [0.212-0.317] | 0.419 | [0.381-0.457] |
| A11 | 0.396 | [0.338-0.453] | 0.410 | [0.353-0.468] | 0.403 | [0.367-0.441] |
| A12 | 0.435 | [0.378-0.493] | 0.385 | [0.327-0.442] | 0.410 | [0.374-0.446] |
| B1 | 0.453 | [0.396-0.511] | 0.615 | [0.558-0.673] | 0.534 | [0.500-0.570] |
| B2 | 0.532 | [0.475-0.590] | 0.439 | [0.381-0.496] | 0.486 | [0.451-0.520] |
| B3 | 0.399 | [0.342-0.457] | 0.612 | [0.554-0.669] | 0.505 | [0.471-0.540] |
| B4 | 0.439 | [0.381-0.500] | 0.482 | [0.425-0.540] | 0.460 | [0.428-0.493] |
| B5 | 0.532 | [0.475-0.590] | 0.291 | [0.237-0.342] | 0.412 | [0.376-0.444] |
| B6 | 0.529 | [0.468-0.590] | 0.277 | [0.227-0.331] | 0.403 | [0.367-0.439] |
| B7 | 0.615 | [0.558-0.673] | 0.237 | [0.187-0.288] | 0.426 | [0.390-0.462] |
| B8 | 0.504 | [0.446-0.565] | 0.245 | [0.194-0.295] | 0.374 | [0.340-0.408] |
| B9 | 0.572 | [0.514-0.630] | 0.126 | [0.090-0.166] | 0.349 | [0.317-0.383] |
| B10 | 0.439 | [0.381-0.500] | 0.522 | [0.464-0.583] | 0.480 | [0.446-0.514] |
| B11 | 0.399 | [0.342-0.457] | 0.586 | [0.529-0.644] | 0.493 | [0.457-0.529] |
| C1 | 0.658 | [0.601-0.721] | 0.176 | [0.133-0.223] | 0.417 | [0.385-0.450] |
| C2 | 0.741 | [0.687-0.791] | 0.205 | [0.158-0.252] | 0.473 | [0.441-0.504] |
| C3 | 0.698 | [0.644-0.752] | 0.122 | [0.086-0.162] | 0.410 | [0.378-0.442] |
| C4 | 0.673 | [0.615-0.727] | 0.155 | [0.112-0.198] | 0.414 | [0.381-0.446] |
| C5 | 0.522 | [0.464-0.583] | 0.259 | [0.209-0.313] | 0.390 | [0.354-0.426] |
| C6 | 0.626 | [0.568-0.684] | 0.133 | [0.095-0.173] | 0.379 | [0.347-0.414] |
| C7 | 0.626 | [0.568-0.684] | 0.133 | [0.094-0.173] | 0.379 | [0.345-0.414] |
| C8 | 0.579 | [0.522-0.637] | 0.119 | [0.083-0.158] | 0.349 | [0.315-0.383] |
| C9 | 0.669 | [0.615-0.723] | 0.119 | [0.083-0.158] | 0.394 | [0.363-0.425] |
| D1 | 0.500 | [0.442-0.558] | 0.388 | [0.331-0.446] | 0.444 | [0.412-0.475] |
| D2 | 0.594 | [0.536-0.651] | 0.331 | [0.277-0.389] | 0.462 | [0.434-0.491] |
| D3 | 0.622 | [0.565-0.680] | 0.104 | [0.068-0.140] | 0.363 | [0.329-0.399] |
| D4 | 0.576 | [0.518-0.633] | 0.266 | [0.216-0.320] | 0.421 | [0.389-0.453] |
| D5 | 0.730 | [0.676-0.781] | 0.205 | [0.158-0.255] | 0.468 | [0.437-0.498] |
| D6 | 0.622 | [0.565-0.680] | 0.241 | [0.191-0.291] | 0.432 | [0.398-0.466] |
| D7 | 0.464 | [0.407-0.522] | 0.547 | [0.489-0.604] | 0.505 | [0.473-0.538] |
| D8 | 0.493 | [0.435-0.550] | 0.500 | [0.442-0.558] | 0.496 | [0.462-0.531] |
| D9 | 0.622 | [0.565-0.680] | 0.295 | [0.241-0.349] | 0.459 | [0.426-0.489] |
| D10 | 0.428 | [0.371-0.486] | 0.097 | [0.065-0.133] | 0.263 | [0.228-0.297] |
| D11 | 0.658 | [0.601-0.712] | 0.094 | [0.061-0.130] | 0.376 | [0.342-0.410] |
